# A Novel *SLC25A4* Variant Causing Mitochondrial Dysfunction, Myopathy and Cardiomyopathy: A Functional and Molecular Characterization

**DOI:** 10.3390/ijms27156978

**Published:** 2026-08-03

**Authors:** Mazhor Aldosary, Hanan AlQudairy, Nourah Alshalan, Mohammad A. Al-Muhaizea, Eman Alobeid, Albandary AlBakheet, Ebtissal Khouj, Aljoharah M. Alharbi, Walaa Alenazi, Hanin R. Omar, Monther Alhamdoosh, Abdullah Alsuwaidan, Hindi Alhindi, Ahmed Alfares, Anas M. Alazami, Stefan T. Arold, Dilek Colak, Robert W. Taylor, Namik Kaya

**Affiliations:** 1 Translational Genomics Department, Genomic Medicine Center of Excellence, King Faisal Specialist Hospital and Research Centre (KFSHRC), Riyadh 12713, Saudi Arabia; mazhord@kfshrc.edu.sa (M.A.); halqudairy@kfshrc.edu.sa (H.A.); noura.saud.alshalan@gmail.com (N.A.); esalobeid@kfshrc.edu.sa (E.A.); abinbakheet@kfshrc.edu.sa (A.A.); amalazami@kfshrc.edu.sa (A.M.A.); 2 Department of Neurosciences, King Faisal Specialist Hospital and Research Centre, Riyadh 11211, Saudi Arabia; mmuhaizea@kfshrc.edu.sa; 3 College of Medicine, Al Faisal University, Riyadh 11533, Saudi Arabia; 4 Personalized Genomics Department, Genomic Medicine Center of Excellence, King Faisal Specialist Hospital and Research Centre, Riyadh 11211, Saudi Arabia; ekhouj@kfshrc.edu.sa (E.K.); abalsuwaidan@kfshrc.edu.sa (A.A.); aalfares@kfshrc.edu.sa (A.A.); 5 Department of Pathology and Laboratory Medicine, King Faisal Specialist Hospital and Research Centre, Riyadh 11211, Saudi Arabia; jharbi@kfshrc.edu.sa (A.M.A.); hal-hindi@kfshrc.edu.sa (H.A.); 6 Computational Genomics Department, Genomic Medicine Center of Excellence, King Faisal Specialist Hospital and Research Centre, Riyadh 11211, Saudi Arabia; walenazi@kfshrc.edu.sa (W.A.); homer@kfshrc.edu.sa (H.R.O.); malhamdoosh@kfshrc.edu.sa (M.A.); 7 KAUST Center of Excellence for Smart Health, Biomedical Sciences Division, King Abdullah University of Science and Technology (KAUST), Thuwal 23955-6900, Saudi Arabia; stefan.arold@kaust.edu.sa; 8 Colak Lab, Research and Innovation, King Faisal Specialist Hospital and Research Centre, Riyadh 11211, Saudi Arabia; dkcolak@gmail.com; 9 Mitochondrial Research Group, Translational and Clinical Research Institute, Faculty of Medical Sciences, Newcastle University, Newcastle upon Tyne NE2 4HH, UK; robert.taylor@newcastle.ac.uk

**Keywords:** *SLC25A4*, ANT1, ATP production, mitochondrial oxygen consumption, cardiomyopathy, myopathy

## Abstract

*SLC25A4*, solute carrier family 25 member 4, gene is a member of the mitochondrial carrier subfamily within the solute carrier protein family. Pathogenic variants in *SLC25A4* are associated with a spectrum of mitochondrial disorders that exhibit variable inheritance patterns and clinical manifestations. Specifically, dominantly inherited variants are typically associated with progressive external ophthalmoplegia with mitochondrial DNA deletions, recessively inherited variants are linked to myopathy and cardiomyopathy, and de novo variants can result in early-onset fatal disease presentations. In this study, we aimed to identify and characterize the disease-causing mutation(s) in a nine-year-old female patient from a consanguineous Saudi family. The patient was asymptomatic until the age of 3 years, when she presented with cardiomyopathy and myopathy. Comprehensive genetic analysis inclusive of whole exome sequencing and segregation analysis using Sanger sequencing identified an *SLC25A4* variant (NM_001151.4: exon 2: c.112-1G>C) as the most likely cause of the disease. To assess transcript-level effects, we performed RT-PCR on RNA extracted from the patient’s cultured lymphoblast cell lines (LCLs) and fibroblast cell lines (FCLs). RT-PCR analysis demonstrated that the variant causes aberrant splicing, resulting in a 6 bp in-frame deletion (p.Gln37_Val38del) in the ANT1 protein. Quantitative RT-PCR demonstrated reduced SLC25A4 transcript levels in both FCLs and LCLs. Quantitative PCR analysis of mitochondrial DNA demonstrated a trend toward increased mtDNA copy number in patient-derived FCLs compared with controls, suggesting a possible compensatory response to mitochondrial dysfunction. Furthermore, Seahorse assays revealed marked reductions in both oxygen consumption rate (OCR) and extracellular acidification rate (ECAR) in patient-derived FCLs compared with controls. These findings expand the molecular and functional spectrum of SLC25A4-associated disease and may inform clinical practice, including genetic interventions such as preimplantation genetic diagnosis, premarital genetic screening, targeted genetic counseling, and cascade testing of at-risk family members.

## 1. Introduction

Myopathies and cardiomyopathies are genetically heterogeneous disorders caused by variants in genes involved in muscle structure, contraction, and energy metabolism. The substantial genetic overlap between skeletal and cardiac muscle disorders explains their frequent coexistence, particularly in conditions affecting mitochondrial function [1]. Genes encoding membrane proteins are increasingly identified in both human and mouse genomes [2]. Solute carriers (SLCs) are one of the important membrane molecules involved in transporting substrates and molecules to their destinations in living cells. To date, more than 400 different membrane-solute carriers organized within 65 families have been identified in humans [3,4,5]. Among the SLC carriers, a subgroup known as SLC25 is critical for substrate transportation into mitochondria and hence indirectly controls critical energy processes [6,7,8]. The SLC25 family contains a large group of mitochondrial carrier proteins, including the ADP/ATP carriers (adenine nucleotide translocases, ANTs). In humans, there are four distinct isoforms of ADP/ATP carriers: SLC25A4 (ANT1; OMIM 103220), SLC25A5 (ANT2; OMIM 300150), SLC25A6 (ANT3; OMIM 300151), and SLC25A31 (ANT4), with different expression levels in tissues [9]. SLC25A4 (ANT1; OMIM 610849) is known as a predominant isoform in the heart and skeletal muscle and also expressed at a low level in proliferating cells and skin cells [10,11], whereas SLC25A5 is highly expressed in regenerative tissues such as the kidney and liver [12,13]. While SLC25A6 is ubiquitously expressed at low baseline levels, SLC25A31 is predominantly expressed in the liver, testis, and brain [12,13]. The differences in tissue specificity are dependent upon the distinct energy requirements of various cell types in different states of cellular differentiation [9,14,15].

*SLC25A4* is located on chromosome 4q35 and contains four exons. *SLC25A4* encodes the ADP/ATP gated pore carrier (translocator), which translocates ADP from the cytoplasm into the mitochondrial matrix and transfers ATP from the mitochondrial matrix into the cytoplasm [16,17]. This carrier forms a homodimer embedded in the inner mitochondrial membrane [18]. Several different mutations in *SLC25A4* have been identified, causing autosomal dominant progressive external ophthalmoplegia (PEOA2) with mitochondrial DNA deletions [19,20,21], autosomal recessive mitochondrial DNA depletion syndrome-12B [22,23,24], and autosomal dominant mitochondrial DNA depletion syndrome-12A cause by de novo dominant-acting variants [17].

Here, we report a novel pathogenic six-base-pair in frame deletion in the exon 1/2 junction (SLC25A4: NM_001151.4: c.112-1G>C: p.Gln37_Val38del) in the *SLC25A4* gene, which caused cardiomyopathy and myopathy in a patient from a consanguineous Saudi family, expanding the genotypic spectrum of SLC25A4 deficiency.

## 2. Results

### 2.1. Clinical Description

**Figure 1:**
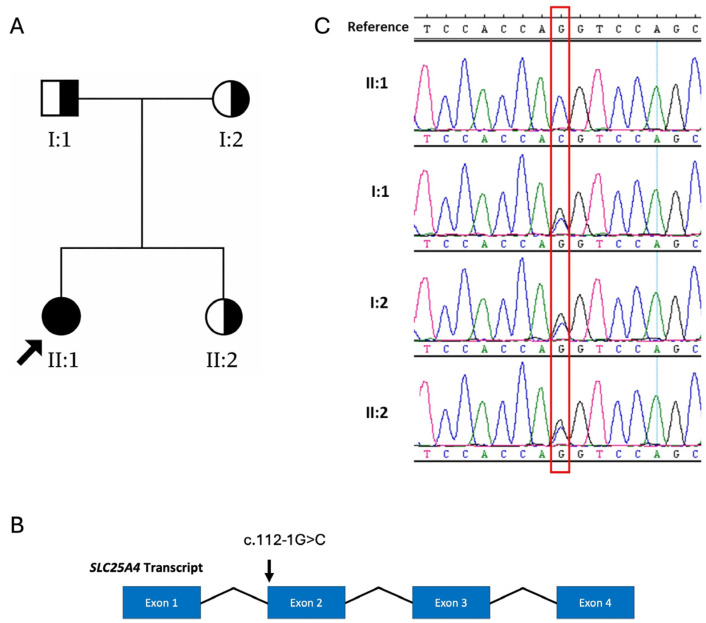
Genetic analysis results. (**A**) Pedigree of the family. The affected individual is represented by the black colored (filled) symbol. Carriers are represented with half-filled symbols. (**B**) Schematic presentation of the human *SLC25A4* transcript showing the four exons and introns (top) and location of the deletion in the gene (bottom). (**C**) The Sanger sequencing chromatograms showing the status of each individual and the segregation of the variant in the family. Individuals are labelled according to their labelled numbers in the pedigree.

The patient is a 9-year-old girl, a product of a full-term, normal, spontaneous vaginal delivery. Her parents are consanguineous (Figure 1), with positive family history for mitochondrial disease. She was asymptomatic until age of 3 to 4 years. She started experiencing easy fatigability from prolonged walking or running. She suffered from swallowing difficulties that forced her to eat small portions frequently. Her growth parameters were within normal ranges. She had no dysmorphic features or seizures. Skin examination revealed hyperpigmentation on the right side of the chest involving the right arm, but no other neurocutaneous stigmata. Physical examination showed proximal motor weakness with decreased reflexes. She had a positive Gower’s sign when getting up from the floor. She had mild ptosis and upgaze ophthalmoplegia. There was no other cranial nerve involvement. Her blood lactic acid levels were high. Echocardiography showed mild ventricular septal wall hypertrophy and posterior wall hypertrophy. She was treated with coQ10 100 mg bid and 1.5 mL carnitine bid and showed clear improvement in her endurance, walking distance and ability to carry her school bag. The patient also reported a clear difference when she stopped the supplement for one month, as she ran out of the supply. The patient is active and independent in her activities of daily living.

### 2.2. Genetics and Molecular Studies

**Figure 2:**
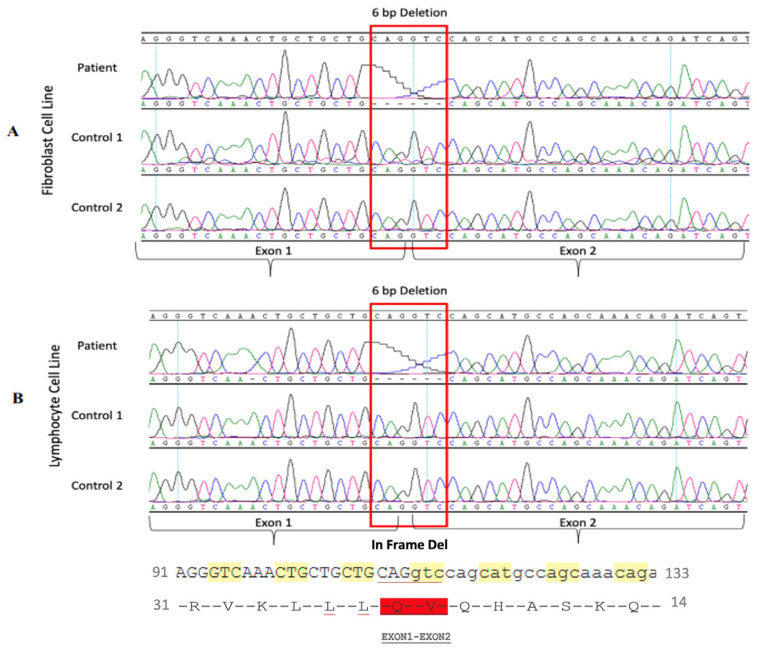
RT-PCR analysis demonstrating a 6 bp in-frame deletion in SLC25A4 cDNA. Sequence chromatograms of cDNA from the patient and two unrelated controls in fibroblast cell lines (FCLs, panel (**A**)) and lymphocyte cell lines (LCLs, panel (**B**)) are shown, with the deleted region highlighted by red boxes. The 6 bp in-frame deletion at the exon 1–exon 2 junction is present only in the patient and absent from the controls (Control 1 and Control 2). Yellow shading denotes individual codons in the exon exon 1, each aligned in-register with its corresponding amino acid in the translated sequence below. While red underlines sequence (the 6 bp) indicates the deleted bases, red shading below marks the amino acids removed by the 6 bp in-frame deletion at the exon 1–exon 2 junction.

Sequencing of the mtDNA was performed for the collected samples (from the index patient) and revealed no pathogenetic variants. To search for likely pathogenic variants in nuclear genes, we carried out WES coupled with autozygosity mapping based on genome-wide SNP calls to detect shared runs of homozygosity (ROHs) in the family, including the index case. Variants in three genes were identified—*BAG3*, *MYH7*, and *SLC25A4*—which filtered down based on previously reported protocols [25,26,27,28,29,30]. Although BAG3 and MYH7 are established disease-associated genes, the identified variants were excluded because they did not segregate with the disease phenotype in a manner consistent with the observed inheritance pattern and lacked sufficient evidence to support pathogenicity according to ACMG/AMP criteria. The analysis singled out a variant (NM_001151.4: exon 2: c.112-1G>C) in *SLC25A4* (Figure 1B). Subsequent RT-PCR analysis demonstrated that this variant causes aberrant splicing, resulting in a 6 bp in-frame deletion at the exon 1/exon 2 junction (c.109_114del), which is predicted to remove two amino acids from the ANT1 protein (p.Gln37_Val38del). The novel variant was fully segregated in the family, being homozygous in the index patient and found as heterozygous in other family members (Figure 1C). The variant was not found in our in-house exomes and local databases such as CGMdb, Saudi Human Genome Database (SHGP) and King Abdullah International Medical Research Center (KAIMRC) Genomic Database (KGD), as well as ethnically matching control data sets (totals to more than 2703). The databases did not have any positive match at the homoallelic state for the variant. Moreover, the variant was not encountered in publicly available international databases and sources such as ClinVar and gnomAD. Based on ACMG/AMP criteria, the variant was classified as pathogenic, supported by its location at a canonical splice acceptor site, segregation with disease, low frequency in population reference databases, and the functional evidence presented in this study. RT-PCR analysis was carried out on the RNA extracted from the patient-derived LCLs and FCLs, which confirmed the predicted 6 bp in-frame deletion (Figure 2A,B).

**Figure 3:**
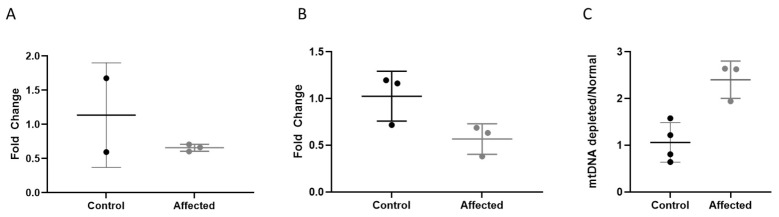
Quantitative RT-PCR analysis and mitochondrial DNA copy number. (**A**,**B**) Relative SLC25A4 transcript levels were determined by quantitative RT-PCR in patient-derived FCLs and LCLs and compared with controls. Expression was reduced to approximately 0.58-fold (42% reduction) in FCLs and 0.55-fold (45% reduction) in LCLs relative to controls; however, these reductions did not reach statistical significance. Control FCLs (*n* = 2), LCLs (*n* = 3); patient (*n* = 1). Quantitative RT-PCR reactions were performed in triplicate for each biological sample. Data are presented as mean ± SD. (**C**) Quantitative PCR analysis of mitochondrial DNA (mtDNA) copy number in patient-derived FCLs (*n* = 1) and control FCLs (*n* = 4) demonstrated a trend toward increased mtDNA copy number in the patient’s FCLs compared with controls (*p* = 0.057). Data are presented as mean ± SD. Technical qPCR reactions were performed in triplicate for each biological sample. Statistical analysis was performed using the two-tailed Mann–Whitney U test.

**Figure 4:**
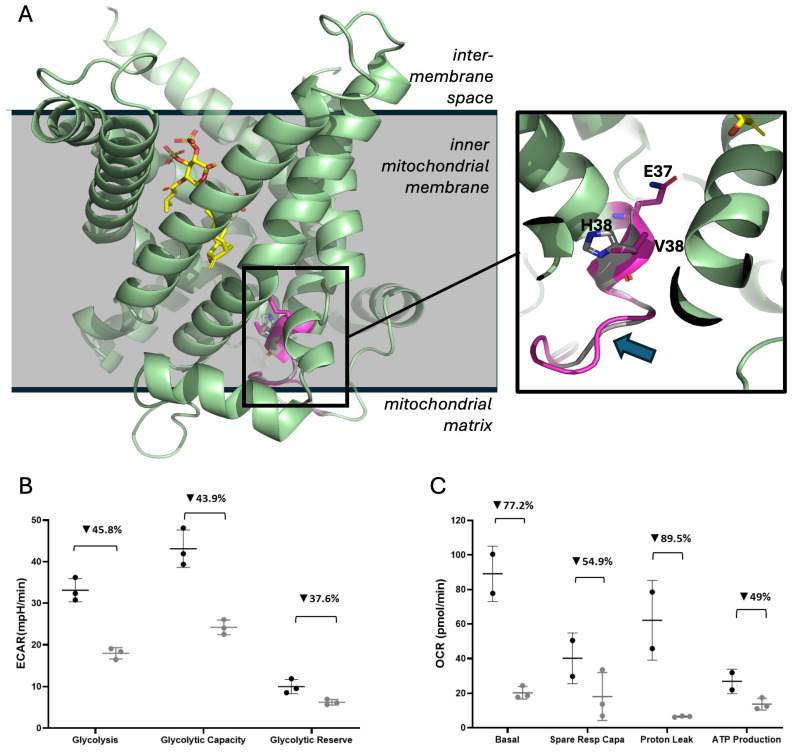
Structural analysis and Seahorse XF metabolic analyses of patient-derived FCLs. (**A**) Structural analysis of the variant. The structures of SLC25A4 (light green ribbon) were modelled based on the 96% identical bovine homologue (PDB 1okc). The region affected by the deletion is shown in cyan and grey for the wild-type and variant, respectively. The inner membrane region is represented by light blue shading. A carboxyatractyloside molecule (taken from 1okc) is shown as a stick model, with carbons and sulfates in yellow and oxygens in red. The zoom panel shows the side chains for the wild-type E37V38 (magenta) and the predicted H38 position in the variant (grey). An arrowhead highlights the effects of the deletion on the loop following residues 38. (**B**,**C**) Seahorse XF metabolic analyses of patient-derived FCLs. Extracellular acidification rate (ECAR) and oxygen consumption rate (OCR) were measured in FCLs from the patient and healthy controls. (**B**) Glycolysis Stress Test showing basal glycolysis, glycolytic capacity, and glycolytic reserve. (**C**) Mitochondrial Stress Test showing basal respiration, spare respiratory capacity, proton leak, and ATP production. Each dot represents one Seahorse assay well. Data are presented as mean ± SD. Seahorse analyses were performed using one patient-derived FCL and one healthy control FCL, with three technical replicate wells analyzed for each condition. Black symbols indicate the control, and light grey symbols indicate the affected individual. Statistical comparisons were performed using the two-tailed Mann–Whitney U test. Exact *p* values are reported in the Results and Supplementary Table S2.

To gain functional insight into the likely consequences due to the 6 base-pair deletion, we performed a quantitative RT-PCR to assess the RNA expression levels of *SLC25A4* in FCLs and LCLs. Compared with controls, *SLC25A4* expression was reduced to approximately 0.58-fold (42% reduction) in FCLs (Figure 3A) and 0.55-fold (45% reduction) in LCLs (Figure 3B). However, these reductions did not reach statistical significance. We next investigated whether the variant was associated with alterations in mitochondrial DNA (mtDNA) copy number in patient-derived FCLs using quantitative PCR. A trend toward increased mtDNA copy number was observed in the patient’s FCLs compared with controls (*p* = 0.057; Figure 3C). Furthermore, we performed structural modeling based on the 96% identical bovine homologue (PDB: 1okc). The analysis suggests that the in-frame deletion may introduce local distortion within a helix–loop element of the transmembrane transporter, potentially affecting the conformational transitions between the inward-open and outward-open states (Figure 4A). Consequently, the variant is predicted to impair ADP/ATP transport activity. Since the XF Seahorse assay is very sensitive [31,32] and the SLC25A4 has some expression in skin/fibroblasts—albeit at low levels—we decided to utilize Seahorse assays to further investigate the likely consequences of the novel (NM_001151.4: exon 2: c.112-1G>C) variant. First, we performed a Seahorse XF Glycolysis Stress Test on patient-derived FCLs. Compared with healthy controls, the patient-derived FCLs exhibited reduced extracellular acidification rates (ECARs), with basal glycolysis, glycolytic capacity, and glycolytic reserve decreased by approximately 45.8% (0.54-fold), 43.9% (0.56-fold), and 37.6% (0.62-fold), respectively (Figure 4B). Similarly, the Seahorse XF Mito Stress Test demonstrated reduced oxygen consumption rates (OCRs), including basal respiration (77.2%; 0.23-fold), spare respiratory capacity (54.9%; 0.45-fold), proton leak (89.5%; 0.11-fold), and ATP production (49.0%; 0.51-fold) in patient-derived FCLs compared with controls (Figure 4C). Although these findings indicate marked impairment of glycolytic and mitochondrial function, the differences did not reach statistical significance using the Mann–Whitney U test, likely reflecting the limited number of biological samples available for analysis.

## 3. Discussion

In humans there are four tissue-specific isoforms of ADP/ATP carriers, with different expression levels in tissues [9]. SLC25A4 (ANT1) is highly expressed in the heart, skeletal muscle, and brain but has low abundance in skin cells, whereas SLC25A5 (ANT2) and SLC25A6 (ANT3) show moderate expression levels in skin at both mRNA and protein levels [33,34]. The fourth family member, SLC25A31 (ANT4), is predominantly expressed in the testis and exhibits very low expression in skin, similar to SLC25A4 [33,34]. SLC25A4 harbors numerous pathogenic variants in both heterozygous and biallelic states, which together underlie a spectrum of mitochondrial diseases in humans [14,17,19,22,23,35,36]. Heterozygous *SLC25A4* mutations cause autosomal dominant progressive external ophthalmoplegia (PEOA2) with multiple mitochondrial DNA deletions and oxidative phosphorylation defects, typically presenting in adulthood with a progressive but usually nonfatal course [14,19,35,36]. In addition, recurrent de novo heterozygous mutations in *SLC25A4* lead to mitochondrial DNA depletion syndrome 12A, characterized by severe early-onset mitochondrial disease with neonatal presentation and often fatal outcomes during infancy [17]. Conversely, biallelic loss-of-function mutations in *SLC25A4* cause autosomal recessive mitochondrial DNA depletion syndrome 12B, which usually manifests in childhood with progressive myopathy and cardiomyopathy, but affected individuals generally survive into adulthood [22,23].

The combined myopathy and cardiomyopathy phenotype observed in our patient has also been reported in other inherited muscle disorders. For example, pathogenic variants in *DES*, encoding the intermediate filament (IF) protein desmin, are known to cause both skeletal myopathy and cardiomyopathy, highlighting the close functional relationship between skeletal and cardiac muscle. Similar overlapping phenotypes have been described in *BAG3*, *FLNC*, and *LMNA*-related disorders [37,38,39,40,41,42,43,44]. The involvement of both tissues in *SLC25A4* deficiency further emphasizes the critical role of mitochondrial energy metabolism in muscle function. To better understand the phenotypic spectrum associated with SLC25A4, several studies have investigated the clinical consequences of both dominant and recessive variants in this gene.

The pioneering study of Kaukonen et al. (2000) [19] revealed heterozygous missense variants in *SLC25A4*, which were identified in five Italian families and a sporadic case, leading to dominantly inherited progressive external ophthalmoplegia and ptosis (PEOA2) but no generalized muscle weakness [17]. A follow-up study by Napoli et al. (2001) described a novel heterozygous *SLC25A4* mutation in a Greek family, expanding genotype-phenotype correlations [20]. The disease is characterized by multiple mitochondrial DNA deletions in skeletal muscle and progressive adult onset usually characterized by exercise intolerance and weakness of the external eye muscles.

In contrast, Palmieri et al. (2005) reported the first recessively inherited homozygous *SLC25A4* mutation in a patient presented with hypertrophic cardiomyopathy, mild myopathy with exercise intolerance and lactic acidosis but no ophthalmoplegia [22]. Later on, Tosserams et al. (2018) [45] reported additional cases of mitochondrial myopathy with similar onset and clinical features highlighted with exercise intolerance, hyperlactatemia and hypertrophic cardiomyopathy, caused by recessive *SLC25A4* mutations [45]. Interestingly, such patients presented with childhood onset. Detailed clinical and laboratory information from all previously reported autosomal recessive *SLC25A4* cases are presented in Supplementary Table S1.

Furthermore, Thompson et al. (2016) reported recurrent de novo heterozygous missense mutations in seven children from six unrelated families [17]. In contrast to PEOA2-associated adulthood onset, these patients presented at birth with severe hypotonia, lactic acidosis, poor or absence of motor development, and respiratory insufficiency. Although, decreased levels of SLC25A4 and several mitochondrial respiratory complexes were observed in the muscle samples, the same defects were not detected in the patient-derived FCLs using Western blot analyses [17]. This study also showed that the mutant proteins were functionally inactive, having low transporter activity, highlighting the important function of affected amino acids [17].

In this study, we report a novel homozygous pathogenic variant (NM_001151.4: exon 2: c.112-1G>C) in *SLC25A4*, which caused cardiomyopathy and myopathy in a patient from a consanguineous Saudi family, expanding the genotypic spectrum of SLC25A4 deficiency. Sequencing analyses revealed that this variant was fully segregated with the phenotype in the family. RT-PCR revealed a 6 bp in-frame deletion in cDNAs generated from the index patient’s LCLs and FCLs. Quantitative RT-PCR demonstrated reduced SLC25A4 transcript levels in patient-derived LCLs and FCLs, corresponding to approximately 45% and 42% reductions, respectively, compared with controls. However, these reductions did not reach statistical significance, likely reflecting the limited number of biological samples available for analysis. Quantitative PCR revealed an increased mtDNA copy number in the patient’s FCLs, which may represent a compensatory cellular response to impaired mitochondrial function. Such increases in mtDNA content have been reported in response to bioenergetic stress and mitochondrial dysfunction and may reflect an attempt to maintain cellular energy production despite defective ANT1-mediated ADP/ATP transport [46,47,48]. Due to a lack of patient-derived muscle samples and given that SLC25A4 is expressed in skin cells led us to directly investigate the mitochondrial functions in the index case’s FCLs using the Seahorse platform. To further investigate the functional consequences of the variant, mitochondrial bioenergetics were assessed using Seahorse XF metabolic assays. Patient-derived FCLs demonstrated marked reductions in both extracellular acidification rate (ECAR) and oxygen consumption rate (OCR), with glycolytic parameters reduced by approximately 38–46% and mitochondrial respiratory parameters by 49–89% compared with controls. Hence, mitochondrial functional assays using the Seahorse platform revealed a decline in both glycolytic and mitochondrial functions.

A limitation of this study was the unavailability of muscle tissue for analysis, resulting in functional investigations being primarily performed in patient-derived FCLs, which may not fully reflect the biology of skeletal and cardiac muscle, the tissues most affected by *SLC25A4* deficiency. Nevertheless, SLC25A4 is expressed at low levels in FCLs, allowing investigation of the molecular consequences of the variant. Since low SLC25A4 expression in fibroblasts may underestimate the defects, future studies including assessment of SLC25A4 protein abundance, ADP/ATP transport activity, and respiratory chain complex assembly will be important to further define the molecular consequences of the variant. Therefore, while the observed defects in mitochondrial and glycolytic function support pathogenicity, caution is warranted when extrapolating these findings to the systemic manifestations of the disease.

An additional limitation is that the functional analyses were performed using cells from a single affected individual, which restricted the number of biological replicates and limits the generalizability of the findings. Subsequent studies involving additional affected individuals with target tissue (muscle biopsy) assessment and independent patient-derived cell lines will be important to further validate these observations.

It is relevant to point out that most studies have primarily used blood or muscle tissue for genetic analyses. Patient-derived FCLs have also been utilized in a few previous studies for various purposes: to assess the tissue specificity of the metabolic defect, to perform genetic analysis (DNA and RNA), to investigate the mechanism of mutation impact (NMD), and to measure the activity of a specific enzyme. For example, the earliest study using patient-derived FCLs was Bakker et al. (1993), who found normal oxidative phosphorylation and lactate/pyruvate metabolism in FCLs of their patient, contrasting with the findings in muscle [49]. Korver-Keularts et al. (2015) used FCLs to demonstrate the involvement of nonsense-mediated decay in the reduced expression of the mutated *SLC25A4* [24]. It is also noteworthy to mention that the Thompson et al. (2016) study did not detect any OXPHOS defect in the FCLs using the WB technique [17]. However, previous mutations reported by this study are mostly de novo heterozygous missense mutations (two different mutations appearing in seven patients, with p.Arg80His occurring in four different unrelated patients).

Our patient has a 6 bp homozygous in-frame deletion at the junction site of exon1/2. The deletion is at a highly conserved position (Supplementary Figure S1) and transmitted through the heterozygous carrier parents. Although, the deletion does not lead to any splicing defect in the patient’s cells, in general, deletions have a more severe effect on protein functions [50,51,52,53,54,55]. Hence it is probable that the deletion could affect SLC25A4’s stability, folding, or interaction with other proteins, leading to impaired function of the transporter. Compensatory mechanisms (e.g., upregulation of other ADP/ATP carriers) may not be sufficient to fully rescue mitochondrial function, especially if the mutation has a severe impact on protein function. While SLC25A4 is highly expressed in heart and skeletal muscle, it is still present in FCLs. The impact of SLC25A4 deficiency might be subtle but detectable under specific stress conditions using Seahorse assays, which are known for their high assay sensitivity. The 6bp deletion could compromise mitochondrial ATP production, affecting OCR and ECAR as measured by Seahorse’s glycolytic stress test. Even if other isoforms (ANT2, ANT3, ANT4) may compensate to some extent in FCLs, they might not fully rescue the defect caused by SLC25A4/ANT1 deficiency, especially under conditions of high energy demand such as a glycolytic stress. Moreover, mitochondria and cellular energy production are under the control of both mitochondrial and nuclear encoded genes [56]. It is possible that a reduction in both OCR and ECAR could be attributed to biochemical changes, which may directly or indirectly interrupt the nuclear and mitochondrial communication, resulting in different expression affecting the cellular function and metabolism [57,58]. Respiration defect is the most common phenotype in patients with mtDNA depletion syndrome due to *SLC25A4* mutations, particularly in congenital cases [17].

## 4. Materials and Methods

### 4.1. Patients

This study was conducted at KFSHRC using an institutional review board-approved protocol (RAC#2120022). The patient and family members (Figure 1A) were asked to sign informed written consents and afterwards were fully apprised of the project.

### 4.2. Sample Collection and Nucleic Acid Isolation

Blood and skin biopsy collections and the generation of patient-derived cell lines were performed according to standard protocols. DNA and RNA extraction were performed using QIAGEN kits (Venlo, The Netherlands). Nucleic Acid quality and quantities were determined via Bioanalyzer 1200 (Agilent Technologies, Santa Clara, CA, USA). Cell passaging and harvesting was done according to standard protocols.

### 4.3. Affymetrix Axiom Human Mapping Assays and Autozygosity Detection

High-density axiom arrays (Affymetrix Inc., Santa Clara, CA, USA) were utilized for genome-wide SNP genotyping. Assays were run on Gene Titan (Affymetrix Inc.). Chip hybridization, scanning, image processing, and preliminary data analysis were all done according to manufacturers’ protocols and guidelines. Autozygome analysis was performed using AutoSNPa software (http://www.insilicase.com/Desktop/autosnpa.aspx, accessed on 1 July 2026) [25,26,29].

### 4.4. Sanger Sequencing

DNA was amplified using SLC25A4 specific primers in separate PCRs. Then, each fragment was sequenced using either forward or reversed primers tagged with universal M13 primers according to standard protocols. The primer sequences (targeting NM_001151.4: exon 2: c.112-1G>C) are as follows:

5′-ACCTTTTCCCTCAAATACCCTAA-3′ (Forward)

5′-CTGCTTGTACTTGTCCTTGAAG -3′ (Reverse)

### 4.5. Whole Exome Sequencing (WES) and Variant Filtering

WES was undertaken using an Illumina 2500 platform. Libraries were prepared by the SureSelect kit (Illumina Inc., San Diego, CA, USA). Captured sequences were read on the system and aligned using an in-house pipeline. The generated data were analyzed using publicly and commercially available tools and software. After the analysis of the WES data, the variants were filtered as previously described [25,26,29,59,60].

### 4.6. mtDNA Sequencing

Genomic DNA was extracted from FCLs and was used as a template for mtDNA amplification. Complete genome amplification, sequencing, and analyses of sequencing results were all performed as detailed elsewhere [61,62].

### 4.7. Quantitative PCR (qPCR)-Based mtDNA Copy Number Assessment

A quantitative assessment of mtDNA copy number was carried out as previously published [25,63]. Genomic DNA extracted from FCLs was used as the starting material. The mitochondrial gene ND1 and the single-copy nuclear gene NALCN were used as mitochondrial and nuclear genome markers, respectively. Relative mtDNA copy number was determined by normalizing ND1 amplification to NALCN amplification using the ΔΔCt method, with control samples serving as the calibrator. NALCN was selected as a stable nuclear reference gene to account for differences in DNA input between samples. Patient-derived FCLs and LCLs were established from the index case. Control samples consisted of four FCLs and three LCLs obtained from healthy male and female donors, including both pediatric and adult individuals. Unless otherwise stated, fibroblast cell experiments were performed using one patient-derived FCLs and four independent control FCLs, while lymphoblast cell experiments were performed using one patient-derived LCLs and three independent control LCLs.

### 4.8. RT-PCR

High quality and undegraded RNA was used for cDNA synthesis using a High-Capacity cDNA Reverse Transcription Kit (Applied Biosystems, Thermo Fisher Scientific, Waltham, MA, USA) as previously published [63,64]. For the primers used in RT-PCR, the Primer3 web tool was utilized for exon spanning primers to ensure the successful and exclusive amplification of exonic regions without the intrusion of genomic sequences.

### 4.9. Quantitative RT-PCR (RT-qPCR)

The cDNA was synthesized from the total RNA utilized for quantitative RT-PCR experiments as previously specified [25,62]. The quantitative expression of *ISCA2* in the patients’ FCLs was analyzed using previously optimized primers. The expression analysis was conducted using the delta delta CT approach [25,62].

### 4.10. Seahorse XFp Metabolic Assays

We used the Seahorse XFp analyzer (Seahorse-Agilent Technologies) to measure the oxygen consumption rate (OCR) and extracellular acidification rate (ECAR), to determine the key parameters of cellular metabolism, using control and patient cells as described [65,66]. One day prior to the assay, FCLs (20,000 cells/well) were seeded in the Seahorse 8-well mini culture plate with growth medium (MEM supplemented with 10% FBS, 1% Penicillin-Streptomycin, and 1% L-Glutamine) and placed in a 37 °C incubator with 5% CO_2_ and allowed to adhere overnight. The sensor cartridge was hydrated in XF calibrant at 37 °C in a non-CO_2_ incubator overnight. The next day, cells were washed twice with warm assay medium, 180 µL of the assay medium was added per well, and the cell culture mini-plate was placed into a 37 °C non-CO_2_ incubator for 45–60 min prior to the assay.

For the glycolysis stress assay, the final concentrations of 10 mM of glucose, 1 µM oligomycin, and 50 mM 2-deoxyglucose (2-DG) were used after preparation in the XF base assay medium [supplemented only with 2 mM glutamine, (pH 7.4)]. While in the mito stress assay, the final concentrations of 1 µM oligomycin, 1 µM FCCP, and 0.5 µM Rotenone/antimycin A (Rot/AA) were used after preparation in the XF base assay medium [supplemented with 1 mM pyruvate, 2 mM glutamine, and 10 mM glucose (pH 7.4)]. Lastly, the stressor mix of oligomycin and FCCP compounds were combined together to be used in the energy phenotype assay. This mix was prepared by diluting 10 µM oligomycin and 1 µM FCCP (final well concentration) in the XF base assay medium [supplemented with 1 mM pyruvate, 2 mM glutamine, and 10 mM glucose (pH 7.4)]. Each individual experiment was done in 3 replicate wells from control and patient FCLs. The results were calculated and produced using the Seahorse Wave software version 2.6.

For these experiments, one patient-derived FCLs and one-healthy-control FCLs were analyzed. Each sample was measured in three replicate wells representing technical replicates. No independent biological replicates were available for the Seahorse experiments.

### 4.11. Computational Structural Analysis of the Deletion

The amino acid sequences of SLC25A4 were retrieved from the UniProt database (ADT1_HUMAN). Three-dimensional structural models of SLC25A4 (wild-type and deletion variant) were generated using both the AlphaFold3 web server (ipTM = 0.49 and pTM = 0.52) [67] and homology modelling (SWISS-MODEL [68]) based on the 96% identical bovine homologue (PDB 1okc). The structural models were manually inspected, and the deletion in SLC25A4 was analyzed using PyMOL TM 3.1.6.1 (pymol.org) following the protocols presented in [69].

### 4.12. Statistical Analysis

Statistical analyses were performed using GraphPad Prism version 10.4.1 (GraphPad Software, Boston, MA, USA). Data are presented as mean ± standard deviation (SD) unless otherwise stated. Statistical comparisons between patient and controls were performed using the two-tailed Mann–Whitney U test. A *p* value < 0.05 was considered statistically significant. Exact *p* values are provided in Supplementary Table S2.

## 5. Conclusions

We note that the family was not cooperative and refused to donate muscle biopsy, which primarily prevented us performing functional studies on the muscle samples. Taken together, the homozygous deletion in *SLC25A4* is a strong candidate in causing the disease in our patient. In conclusion, our study expands the genotypic spectrum of *SLC25A4*-associated human diseases. Moreover, the use of Seahorse assays on FCLs from those patients with *SLC25A4*-associated diseases may still be useful and informative; however, the results must be interpreted with prudence, especially in the absence of skeletal muscle biopsy data.

## Data Availability

The datasets for this article are not publicly available due to concerns regarding participant/patient anonymity. Requests to access the datasets should be directed to the corresponding author.

## Supplementary Materials

The following supporting information can be downloaded at: https://www.mdpi.com/article/10.3390/ijms27156978/s1. References [22,23,24,45,49,70,71] are cited in the Supplementary Materials.
