# Supplementary material for: A Novel SLC25A4 Variant Causing Mitochondrial Dysfunction, Myopathy and Cardiomyopathy: A Functional and Molecular Characterization
Source: Int J Mol Sci. 2026 Aug 3;27(15):6978. doi: 10.3390/ijms27156978 (PMC13466473; doi:10.3390/ijms27156978)
Supplement: Supplementary file 1 [file ijms-27-06978-s001.zip › Supplementary Table S1-Final.pdf]

Supplementary Table S1. Summary of statistical analyses

Assay/parameter	Control mean ± SD	Patient mean ± SD	Fold change	Change	Primary statistical note
qRT-PCR FCL	1.136 ± 0.763	0.658 ± 0.051	0.58-fold	42.1% reduction	Mann-Whitney U P = 0.99
qRT-PCR LCL	1.026 ± 0.267	0.568 ± 0.163	0.55-fold	44.7% reduction	Mann-Whitney U P = 0.1
mtDNA copy number	1.062 ± 0.427	2.405 ± 0.400	2.26-fold	126.5% increase	Mann-Whitney U P = 0.057
ECAR basal glycolysis	33.109 ± 2.782	17.952 ± 1.354	0.54-fold	45.8% reduction	Mann-Whitney U P = 0.100
ECAR glycolytic capacity	43.112 ± 4.509	24.193 ± 1.771	0.56-fold	43.9% reduction	Mann-Whitney U P = 0.100
ECAR glycolytic reserve	10.003 ± 1.731	6.241 ± 0.662	0.62-fold	37.6% reduction	Mann-Whitney U P = 0.100
OCR basal respiration	89.068 ± 16.030	20.264 ± 3.612	0.23-fold	77.2% reduction	Mann-Whitney U P = 0.200
OCR spare respiratory capacity	40.127 ± 14.574	18.105 ± 13.802	0.45-fold	54.9% reduction	Mann-Whitney U P = 0.400
OCR proton leak	62.118 ± 23.107	6.531 ± 0.353	0.11-fold	89.5% reduction	Mann-Whitney U P = 0.200
OCR ATP production	26.950 ± 7.077	13.733 ± 3.370	0.51-fold	49.0% reduction	Mann-Whitney U P = 0.200

Values are presented as mean ± SD. Fold changes are expressed as patient/control. Relative qRT-PCR expression was calculated using the $2^{-\Delta\Delta Ct}$ method. Statistical comparisons for Seahorse assays and mtDNA copy number were performed using the two-tailed Mann–Whitney U test. qRT-PCR analyses are presented descriptively because only one patient-derived biological sample was available for each cell type.
